# Celiac Disease Emerging After ASCT in Multiple Myeloma: A Rare Diagnostic Intersection and Literature Review

**DOI:** 10.1155/crh/8921605

**Published:** 2026-09-02

**Authors:** Mais Musleh, Mahmoud Alhamadeh Alswij, Qossay Alhusein

**Affiliations:** ^1^ Department of Hematology, Faculty of Medicine, Al Mouwasat University Hospital, Damascus, Syria; ^2^ Department of Internal Medicine, Hematology Department, Al Mouwasat University Hospital, Damascus, Syria

**Keywords:** autologous stem cell transplantation, celiac disease, chronic diarrhea, multiple myeloma

## Abstract

**Background:**

Autologous stem cell transplantation (ASCT) is a standard component of therapy for eligible patients with multiple myeloma. Persistent gastrointestinal symptoms after ASCT are commonly attributed to infection, medication‐related toxicity, or disease relapse, whereas autoimmune enteropathies such as celiac disease are rarely considered.

**Case Presentation:**

We report a 53‐year‐old woman with lambda light‐chain multiple myeloma who achieved complete remission after bortezomib, lenalidomide, and dexamethasone induction followed by ASCT and lenalidomide maintenance. Four months after transplantation, she developed persistent diarrhea and a 4‐kg unintentional weight loss. Infectious investigations were negative, and imaging and bone marrow assessment showed no evidence of myeloma relapse. Upper gastrointestinal endoscopy demonstrated duodenal villous atrophy, and biopsy findings were consistent with Marsh 3A celiac disease. Serum antitissue transglutaminase IgA was markedly elevated at > 123 U/mL. A strict gluten‐free diet resulted in complete resolution of diarrhea within 2 weeks and a subsequent decline in celiac serologic markers. Lenalidomide‐associated diarrhea or enteropathy was considered but could not be definitively excluded because treatment withdrawal and rechallenge were not performed. In addition, pre‐ASCT celiac serology and HLA‐DQ2/DQ8 typing were unavailable.

**Conclusion:**

This case highlights celiac disease as a rare but clinically important cause of persistent diarrhea after ASCT. Immune reconstitution following transplantation may unmask previously subclinical autoimmune disease. Clinicians should maintain a broad differential diagnosis for posttransplant gastrointestinal symptoms, as early recognition and appropriate dietary intervention can lead to rapid symptom resolution and prevent unnecessary treatment interruption or morbidity.

## 1. Background

Multiple myeloma (MM) is a malignant plasma cell disorder characterized by clonal proliferation of plasma cells in the bone marrow, leading to excessive production of monoclonal immunoglobulins and associated complications such as osteolytic bone lesions, anemia, renal dysfunction, and immunosuppression [1]. Autologous stem cell transplantation (ASCT) remains a central component of therapy for eligible patients, offering improved progression‐free survival and quality of life, although it is not curative.

Celiac disease is a chronic immune‐mediated enteropathy triggered by dietary gluten in genetically susceptible individuals, resulting in small intestinal mucosal injury, malabsorption, and a range of gastrointestinal and systemic symptoms [2]. While the coexistence of MM and celiac disease is uncommon, immune dysregulation following ASCT may unmask latent or subclinical autoimmune conditions, including celiac disease [3, 4]. Posttransplant gastrointestinal symptoms are often attributed to infections, medications, or disease relapse, potentially delaying the diagnosis of autoimmune causes.

Here, we report biopsy‐confirmed Marsh 3A celiac disease diagnosed 4 months after ASCT in a patient with lambda light‐chain MM. This case highlights the need for clinicians to maintain a broad differential diagnosis and consider autoimmune enteropathies in transplant recipients presenting with persistent gastrointestinal complaints.

## 2. Case Presentation

A 53‐year‐old female presented with fatigue, unintentional weight loss, and bone pain, complicated by a spontaneous fracture of the right forearm. She had no significant past medical history. Physical examination revealed pallor and swelling localized to the right forearm.

Laboratory investigations showed the following: complete blood count with white blood cells 5 × 10^9^/L, hemoglobin 9.7 g/dL, and platelets 340 × 10^9^/L; LDH 230 U/L, serum iron 119 μg/dL, total iron‐binding capacity (TIBC) 306 μg/dL, and ferritin 32 ng/mL; vitamin B12 350 pg/mL; erythrocyte sedimentation rate (ESR) 35 mm/h; serum creatinine 0.6 mg/dL; and corrected serum calcium 9.3 mg/dL. Serum protein electrophoresis (SPEP) revealed no monoclonal spike. Immunoglobulin quantification showed IgG 957 mg/dL (normal 700–1600 mg/dL), IgA 47 mg/dL (normal 70–400 mg/dL), and IgM 48 mg/dL (normal 40–230 mg/dL). Beta‐2 microglobulin was elevated at 3.5 mg/L. Serum free light chain analysis demonstrated kappa 244 mg/L and lambda 138 mg/L, with a kappa/lambda ratio of 1.3. Twenty‐four‐hour urine protein was elevated at 2180 mg (normal < 150 mg/24 h) with elevated lambda light chain in immunofixation in urine.

**FIGURE 1:**
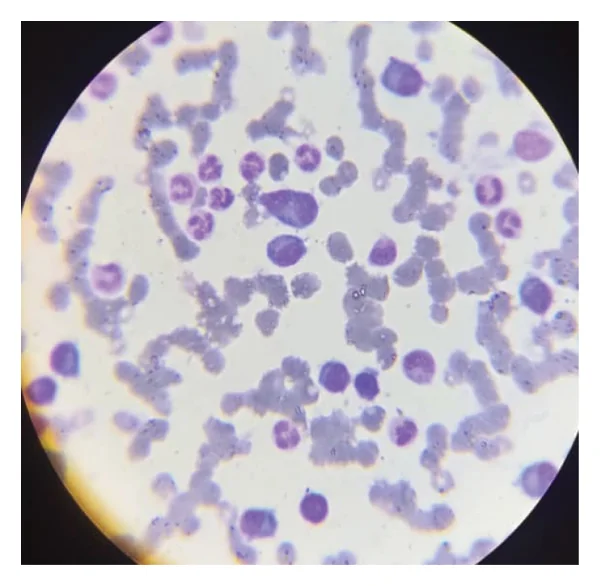
Bone marrow aspirate showed 80% cellularity with 50% plasma cells infiltration (Giemsa stain × 100).

Bone marrow aspirate was hypercellular (80% cellular) with 50% plasma cells as shown in Figure 1. Trephine biopsy confirmed 50% plasma cell infiltration. Immunohistochemistry was positive for CD138 (60%) and lambda light chain, while negative for kappa light chain. No cytogenetic abnormalities were detected.

Positron emission tomography–computed tomography (PET‐CT) demonstrated multiple lytic bone lesions with standardized uptake values (SUVs) of 4–5.

The patient received four cycles of bortezomib, lenalidomide, and dexamethasone (VRD): bortezomib 1.3 mg/m^2^ on Days 1, 8, 15, and 22; lenalidomide 25 mg daily on Days 1–21; and dexamethasone 40 mg once weekly. Zoledronic acid 4 mg was administered monthly. Daratumumab was not available at the treating institution because of medication‐supply constraints related to the ongoing crisis in Syria; therefore, an anti–CD38‐containing quadruplet could not be used. After induction, hemoglobin increased to 11.8 g/dL, 24‐h urinary protein decreased to 94 mg, beta‐2 microglobulin decreased to 2.4 mg/L, and the patient met criteria for complete remission, although measurable residual disease was reported at 0.2%.

She underwent ASCT after high‐dose melphalan conditioning at 200 mg/m^2^. A total of 8 × 10^6^ CD34‐positive cells/kg were infused. The immediate posttransplant course was uncomplicated apart from mild oral mucositis, and no infection was documented. Lenalidomide maintenance at 10 mg daily was initiated and continued during follow‐up.

Four months posttransplant, the patient developed chronic diarrhea (4 episodes daily) with a 4 kg weight loss over 1 month. Laboratory investigations revealed hemoglobin 11.1 g/dL, ESR 14 mm/h, creatinine 0.8 mg/dL, corrected calcium 8.9 mg/dL, IgG 1600 mg/dL, IgA 184 mg/dL, IgM 74 mg/dL, kappa 390 mg/L, lambda 181 mg/L, and a kappa/lambda ratio of 1.2. C‐reactive protein was 0.5 mg/dL (within normal limits). PET‐CT showed no evidence of active disease. Bone marrow aspirate and biopsy showed no plasma cells, and MRD was undetectable.

Given the persistence of gastrointestinal symptoms, a comprehensive infectious evaluation was performed to exclude common posttransplant infections. Polymerase chain reaction (PCR) assays for cytomegalovirus (CMV) and Epstein–Barr virus (EBV) were negative, and HIV serology was also negative. Pre‐ASCT celiac serology had not been obtained, and HLA‐DQ2/DQ8 typing was not performed.

**FIGURE 2:**
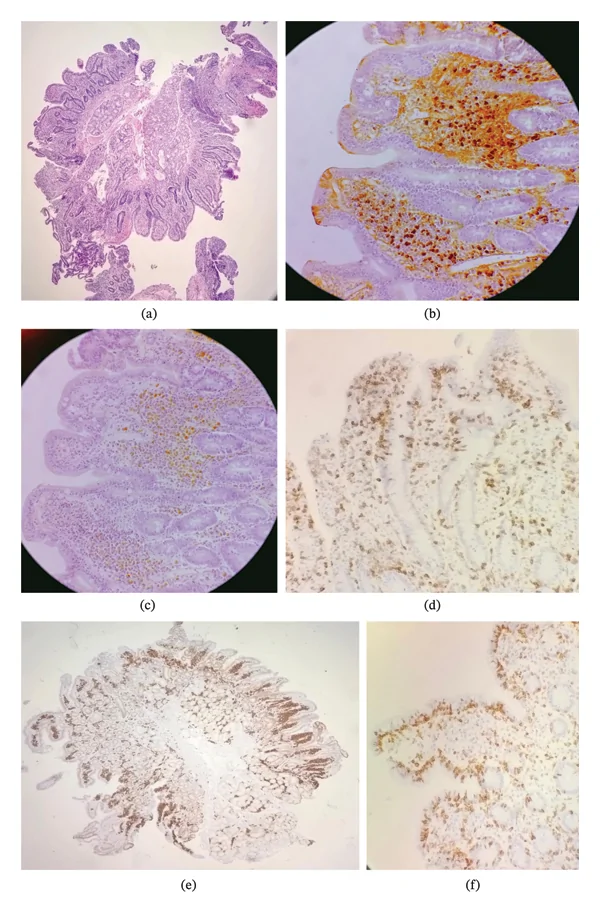
Duodenal biopsy showed increased IELs, hypertrophic crypts, atrophic villi, and mild partial villous atrophy, which was classified with Marsh 3A (a). Mild increase in IE T lymphocytes in CD03 and CD05 as shown in (e, f). Abundant increase in CD138 as shown in (d). Negative for kappa and lambda as shown in (b, c).

Upper gastrointestinal endoscopy subsequently demonstrated duodenal villous atrophy. Histopathological examination of biopsy specimens demonstrated features consistent with celiac disease, classified as Marsh 3A. Immunohistochemical staining showed a mild increase in intraepithelial CD3+ and CD5+ T lymphocytes, alongside abundant CD138+ plasma cells. Light chain immunostaining was negative for both kappa and lambda chains, as shown Figure 2. Serological testing revealed markedly elevated immunoglobulin A anti–tissue transglutaminase (IgA‐tTG) antibodies > 123 U/mL (reference range: < 12 U/mL), supporting the diagnosis of celiac disease.

Following initiation of a strict gluten‐free diet, the patient experienced rapid clinical improvement, with complete resolution of diarrhea and normalization of stool frequency within 2 weeks. Repeat serologic testing at 3 months demonstrated a marked decline in anti–tissue transglutaminase IgA levels, supporting dietary adherence and mucosal recovery. Nutritional parameters, including iron, vitamin D, and serum albumin, improved progressively with appropriate supplementation.

Lenalidomide‐associated diarrhea was considered in the differential diagnosis; however, the presence of duodenal villous atrophy, positive celiac serology, and the prompt clinical response to a gluten‐free diet favored celiac disease as the underlying etiology. The patient remained in complete remission from MM, with no evidence of disease relapse or transplant‐related complications at 24 months following ASCT.

## 3. Discussion

MM is a malignant plasma cell neoplasm characterized by the clonal proliferation of plasma cells, typically producing monoclonal immunoglobulin or free light chains. This uncontrolled expansion often leads to skeletal destruction, anemia, hypercalcemia, and renal dysfunction [1]. In our case, the diagnosis was confirmed by 50% marrow plasma cells, osteolytic lesions, and elevated free light chains.

The standard therapeutic approach for transplant‐eligible patients includes induction therapy with a triplet regimen comprising a proteasome inhibitor, immunomodulatory agent, and corticosteroid (e.g., VRD), followed by ASCT and maintenance therapy, most commonly with lenalidomide [5]. Our patient achieved complete remission after VRD induction and ASCT, with undetectable MRD during follow‐up. Daratumumab was not added to induction because it was not available in the treating center owing to limitations related to the ongoing situation in Syria. Although a daratumumab‐containing regimen would have been preferred if accessible, VRD represented the most feasible induction strategy in this setting.

However, the unexpected development of persistent diarrhea and significant weight loss in the early posttransplant period prompted a thorough gastrointestinal workup. In transplant recipients, gastrointestinal symptoms are commonly attributed to infection, medication toxicity, or disease relapse. However, extensive infectious workup and reassessment of disease status excluded these possibilities. Endoscopic evaluation revealed villous atrophy consistent with Marsh 3A, along with increased intraepithelial lymphocytes—findings diagnostic of celiac disease.

Celiac disease is an immune‐mediated enteropathy triggered by gluten ingestion in genetically susceptible individuals [2]. Although the coexistence of celiac disease and MM is exceedingly rare, the clinical overlap is noteworthy. Celiac disease may result in malabsorption, nutritional deficiencies, impaired absorption of orally administered drugs, and delayed recovery after transplantation. It is also associated with an increased risk of selected gastrointestinal and lymphoproliferative malignancies. Nevertheless, an association between celiac disease and MM remains rarely reported [6, 7].

The temporal association between ASCT and the onset of gastrointestinal symptoms raises the possibility that posttransplant immune reconstitution contributed to the clinical emergence of previously unrecognized celiac disease. However, a causal relationship cannot be established in this case. Pretransplant celiac serology was not available, and HLA‐DQ2/DQ8 testing was not performed. Therefore, it is not possible to determine whether celiac disease was already present in a subclinical form before transplantation or developed de novo afterward. The term “unmasking” should consequently be interpreted as a possible explanation rather than a proven mechanism.

Lenalidomide‐associated diarrhea or enteropathy was also considered. This possibility could not be excluded definitively because lenalidomide was not withdrawn and subsequently reintroduced, and no formal drug rechallenge was performed. Nevertheless, the presence of characteristic duodenal villous atrophy, positive celiac serology, and prompt resolution of symptoms after initiation of a strict gluten‐free diet favored celiac disease as the principal explanation. The absence of plasma cell infiltration and light‐chain restriction in the duodenal biopsy, together with negative imaging and bone marrow findings, also argued against gastrointestinal myeloma relapse or extramedullary disease.

Only a limited number of reports have described celiac disease after hematopoietic cell transplantation. One report described celiac disease diagnosed 2.5 years after ASCT in a patient with acute myeloid leukemia [4], while other publications have documented either coexistence of MM and celiac disease or changes in celiac disease activity after transplantation [8–10]. To our knowledge, the present case is among the earliest reported presentations of biopsy‐confirmed celiac disease after ASCT in a patient with MM, with symptoms beginning 4 months after transplantation. This observation should, however, be interpreted cautiously because the absence of baseline serology prevents confirmation of new‐onset disease.

Published reports suggest that hematopoietic cell transplantation may have variable effects on celiac disease. In some cases, allogeneic transplantation has been followed by remission of preexisting celiac disease, possibly because of immune reconstitution [10]. Conversely, de novo celiac disease has been described after allogeneic transplantation, particularly when stem cells were obtained from HLA‐matched sibling donors with celiac disease [3, 4]. These contrasting observations indicate that transplantation may influence immune tolerance in different ways depending on host susceptibility, donor characteristics, conditioning, and posttransplant immune recovery. However, the mechanisms remain incompletely understood, particularly after autologous transplantation, where adoptive transfer of donor immune memory does not occur.

Autoimmune diseases are characterized by dysregulated T‐ and B‐cell responses against self‐antigens and may produce chronic inflammatory stimulation. MM arises from postgerminal‐center B cells that have undergone somatic hypermutation within immunoglobulin variable‐region genes. Chronic antigenic stimulation has therefore been proposed as a potential contributor to plasma cell dyscrasias, although direct causality between celiac disease and MM has not been established [11]. In the present case, the available evidence does not permit conclusions regarding whether celiac‐related immune activation contributed to the development of MM.

This case has several limitations. Pre‐ASCT celiac serology was unavailable, HLA‐DQ2/DQ8 typing was not performed, lenalidomide‐induced enteropathy was not assessed by drug withdrawal or rechallenge, and the follow‐up period of 12 months was relatively short. These limitations restrict conclusions regarding causality, disease onset, and long‐term outcome.

Despite these limitations, this case emphasizes that celiac disease should be considered in patients with persistent diarrhea after ASCT, particularly after infectious causes, medication toxicity, and disease relapse have been investigated. Early endoscopic and serologic evaluation may prevent diagnostic delay, unnecessary interruption of antimyeloma therapy, and progression of nutritional complications. Close collaboration between hematology, gastroenterology, pathology, and nutrition specialists is important in the management of such complex posttransplant presentations.

## 4. Conclusion

This case illustrates a rare but clinically relevant presentation of celiac disease emerging shortly after ASCT in a patient with MM. Persistent gastrointestinal symptoms in the posttransplant setting should prompt consideration of autoimmune enteropathies once infectious and malignant causes have been excluded. Early recognition and dietary intervention can lead to rapid symptom resolution and may prevent unnecessary treatment interruptions or morbidity during maintenance therapy.

**Nomenclature**
ASCT: Autologous stem cell transplantation
CD: Celiac disease
CMV: Cytomegalovirus
CR: Complete remission
EBV: Epstein–Barr virus
HCT: Hematopoietic cell transplantation
MM: Multiple myeloma
MRD: Measurable residual disease
PET‐CT: Positron emission tomography–computed tomography
R‐ISS: Revised International Staging System
VRD: Bortezomib, lenalidomide, and dexamethasone

## Author Contributions

Mais Musleh: design of the study, data collection, data interpretation and analysis, drafting, and critical revision.

Mahmoud Alhamadeh Alswij: data interpretation and analysis, drafting, and critical revision.

Qossay Alhusein: data interpretation and analysis, drafting, and critical revision.

Qossay Alhusein is the guarantor of this work.

## Funding

No funding was required.

## Disclosure

All authors gave approval for the final manuscript.

## Ethics Statement

Ethical approval was not required for this case report.

## Consent

Written informed consent was obtained from the patient for publishing this case report and any accompanying images. A copy of the written consent is available for review by the editor‐in‐chief of this journal on request.

## Conflicts of Interest

The authors declare no conflicts of interest.

## Data Availability

The data that support the findings of this study are available on request from the corresponding author. The data are not publicly available due to privacy or ethical restrictions.
